# FAIR-EuMon: a FAIR-enabling resource for biodiversity monitoring schemes

**DOI:** 10.3897/BDJ.12.e125132

**Published:** 2024-08-01

**Authors:** Juliana Menger, Barbara Magagna, Klaus Henle, Alexander Harpke, Mark Frenzel, Johannes Rick, Karen Wiltshire, Annegret Grimm-Seyfarth

**Affiliations:** 1 Helmholtz Centre for Environmental Research – UFZ, Department of Conservation Biology and Social-Ecological Systems, Leipzig, Germany Helmholtz Centre for Environmental Research – UFZ, Department of Conservation Biology and Social-Ecological Systems Leipzig Germany; 2 Alfred-Wegener-Institut Helmholtz-Zentrum für Polar- und Meeresforschung, Wattenmeerstation Sylt, Sylt, Germany Alfred-Wegener-Institut Helmholtz-Zentrum für Polar- und Meeresforschung, Wattenmeerstation Sylt Sylt Germany; 3 GO FAIR Foundation, Leiiden, Netherlands GO FAIR Foundation Leiiden Netherlands; 4 Helmholtz Centre for Environmental Research – UFZ, Department of Community Ecology, Halle, Germany Helmholtz Centre for Environmental Research – UFZ, Department of Community Ecology Halle Germany

**Keywords:** research data management, metadata form, FAIR, biodiversity monitoring, provenance model, semantic model, metadata schema

## Abstract

**Background:**

Within the scope of the Helmholtz Metadata Collaboration (HMC), the ADVANCE project – Advanced metadata standards for biodiversity survey and monitoring data: supporting of research and conservation – aimed at supporting rich metadata generation with interoperable metadata standards and semantic artefacts that facilitate data access, integration and reuse across terrestrial, freshwater and marine realms. HMC's mission is to facilitate the discovery, access, machine-readability, and reuse of research data across and beyond the Helmholtz Association.

**New information:**

We revised, adapted and expanded existing metadata schemas, vocabularies and thesauri to build a FAIR metadata schema and a metadata entry form built on it for users to provide their metadata instances focused on biodiversity monitoring data. The schema is FAIR because it is both machine-interpretable and follows domain-relevant community standards. This report provides a general overview of the project results and instructions on how to access, re-use and complete the metadata form.

## Introduction

In an ever-changing world, field surveys, inventories and monitoring data are essential to predict biodiversity responses to global drivers, such as land-use and climate change. This understanding builds the basis to timely conservation management. However, due to funding constraints, biodiversity data are usually collected over short periods of time, hampering analyses of long-term trends and predictions of changes. To overcome this issue, biodiversity researchers rely on the integration of datasets collected by different projects, over distinct temporal and spatial scales (Henry et al. 2008, Lengyel et al. 2018). Since field biodiversity data collected across ecosystems can be highly complex and heterogeneous (Lengyel et al. 2018), the successful integration and reuse of such data depend on how they follow the FAIR guiding principles for scientific data management (i.e., Findability, Accessibility, Interoperability, Reusability; Wilkinson et al. (2016))

The FAIR principles do not impose any specific technological implementations, but provide guidance for improving findability, accessibility, interoperability and reusability of digital assets. As such, interpretations of the FAIR principles are needed to support convergence towards consistent FAIR implementations (Jacobsen et al. 2020). Domain-specific communities may, therefore, either choose to reuse existing implementation solutions, to adapt or to create the needed solution, which can be reused again by other communities in the future (Jacobsen et al. 2020). Here, we adopt the FAIR interpretations of the Helmholtz Metadata Collaboration (HMC), whose mission is to facilitate the discovery, access, machine-readability, and reuse of research data across and beyond the Helmholtz Association (Buttigieg 2022). Nonetheless, it is important to highlight that the HMC interpretations have been built upon previous interpretations of other international groups, such as the GO FAIR Foundation (Jacobsen et al. 2020) and the Research Data Alliance (FAIR Data Maturity Model Working Group 2020).

The ADVANCE project aimed at developing a FAIR metadata schema to support the integration and reuse of European biodiversity monitoring data across terrestrial, freshwater and marine ecosystems. This metadata schema (hereafter FAIR-EuMon) is focused on collecting information about existing biodiversity monitoring efforts and their activities, their coverage and data availability. It provides metadata fields that allow the identification of the monitoring schemes, contact information, funding, intellectual rights and data accessibility, as well as monitoring scheme characteristics, such as data collection description, spatial, temporal, taxonomic and habitat coverage (for details see the section *FAIR-EuMon metadata elements and fields* below). Moreover, it uses a user-friendly interface built on top of a machine-actionable format.

The FAIR-EuMon metadata schema was built upon the largest and most comprehensive metadata catalogue for biodiversity monitoring in Europe, which compiled biodiversity monitoring schemes across the continent (EuMon: EU-wide monitoring methods and systems of surveillance for species and habitats of community interest; Henry et al. (2008), Lengyel et al. (2008)). Information on habitat and species monitoring schemes were collected via a questionnaire containing 43 questions sent to coordinators of monitoring schemes, ministry officials and representatives of other stakeholder groups involved in monitoring in all European countries (Lengyel et al. 2008). It resulted in a metadata catalogue of 650+ monitoring schemes publicly available for more than 15 years (DaEuMon; Henle et al. (2010), Henle (2012)). However, due to funding constraints and the need for new software, this valuable database could not be maintained and, as of today, is out of service. Moreover, the FAIR principles had not been published by the time of its creation, so that the EuMon questionnaire and database are not findable, accessible, interoperable nor reusable.

The FAIR-EuMon metadata schema is, therefore, a FAIRification of the EuMon questionnaire. We revised, adapted and expanded the EuMon questionnaire to build a metadata form which is both machine-readable and follows domain-relevant community standards. We relied on existing metadata and semantic standards (ex.: Schema.org, DCAT, Darwin Core), linked open vocabularies (ex. BioPortal Ontologies), persistent identifiers (ex. ORCID, ROR ID, PURL), machine-readable formats (ex. JSON-LD, RDF) and other FAIR Supporting Resources (ex. nanopublications) to rebuild the EuMon metadata schema, resulting in the FAIR-EuMon metadata schema and a metadata entry form.

**Benefits of filling out the FAIR-EuMon metadata form as demonstrated by the EuMon legacy**

Filling out the FAIR-EuMon metadata form on CEDAR requires some effort, which depends on whether monitoring schemes have been already internally documented. If so, it takes around 20 minutes to fill out all the metadata fields. We have not specified optionality indications, so that all fields are to be filled, enriching the metadata instance. This effort is rewarded by a range of benefits that it provides to biodiversity monitoring and conservation, but also to the monitoring schemes themselves. Based on the EuMon database of monitoring schemes, Lengyel et al. (2018) provided indicators for sampling design, sampling effort and data analysis of biodiversity monitoring that can be used by monitoring schemes to explore options for improving their monitoring activities. Likewise, the information on running costs of monitoring schemes included in the metadata form can help planning extensions or the establishment of monitoring schemes. In addition, individuals and organisations engaged in monitoring activities benefit from an increased awareness and legitimacy of their activity with a better recognition of their role as major data providers for biodiversity assessments (Henle et al. 2010). For instance, 72% of the French monitoring schemes entered in the EuMon database were run by volunteer naturalists, adding a high value of citizen scientists for biodiversity monitoring (Schmeller et al. 2009).

For biodiversity conservation and policies, the inclusion of monitoring schemes in metadata databases facilitates the use of synergies amongst monitoring activities targeting different policies. The EuMon database has been used to identify gaps in biodiversity monitoring for plants and habitats listed in European Directives (Lengyel et al. 2008, Kull et al. 2008). Similarly, Mihoub et al. (2017) showed that the temporal baseline for biodiversity may seriously underestimate anthropogenic pressures because most biodiversity monitoring schemes started after anthropogenic pressures had already reached half of their current magnitude. Yet, the inclusion of monitoring schemes in appropriate FAIR databases would substantially increase their potential contribution to biodiversity assessments, overcoming the fact that current data collections do not have sufficient FAIR data to be considered for inclusion in large-scale assessments (Turner et al. 2022).

## Project description

### Title

ADVANCE project – Advanced metadata standards for biodiversity survey and monitoring data: supporting of research and conservation

### Funding

The ADVANCE project (ZT-I-PF-3-025 / RA-269/21) was funded by the Initiative and Networking Fund of the Helmholtz Association within the framework of the Helmholtz Metadata Collaboration project call.

## Web location (URIs)

Homepage: https://www.ufz.de/index.php?en=48268

## Repository

Type: CEDAR

Browse URI: https://doi.org/10.60745/ng90-tp91

Location: https://cedar.metadatacenter.org/dashboard?folderId=https:%2F%2Frepo.metadatacenter.org%2Ffolders%2F909ec64b-8a57-401a-a98a-812d7af2494b

## Usage licence

### Usage licence

Creative Commons Public Domain Waiver (CC-Zero)

## Implementation

### Implements specification

**The FAIR-EuMon metadata template**

We used the CEDAR Workbench (Musen et al. 2015) to implement the FAIR-EuMon metadata schema as a CEDAR template. CEDAR is a free, open source, online platform that centres on the use of metadata templates that define the elements needed to describe particular types of data. CEDAR templates are represented in JSON, which can achieve machine-interpretability by enriching the expressivity with semantics (JSON-LD). The CEDAR environment is linked to BioPortal, an ontology repository with 1130 artefacts (Whetzel et al. 2011, Salvadores et al. 2013), which allow us to feed metadata templates and instances with concepts from semantic artefacts. SKOS vocabularies uploaded in BioPortal are used to create controlled lists and autocomplete functions to minimise typos and spelling errors, while safeguarding consistency in term definitions, and to provide semantic interoperability (Musen et al. 2022).

The FAIR-EuMon metadata template is available online under the CC BY 4.0 licence, and can be accessed and filled out by creating a CEDAR account. For instructions on how to create a CEDAR account or metadata templates, readers may refer to the CEDAR User Guide. Here, we provide an overview of the features of the FAIR-EuMon metadata schema as well as general instructions on how to complete the form.

The metadata form is human-readable, i.e., it appears as a user-friendly, online questionnaire. Its questions (fields) are grouped into sections (elements) and it includes a *read and understood* check box that provides instructions on how to fill in the form. At the same time, it is also FAIR, as its metadata fields are specified as ontological properties from standards, such as Darwin Core, Dublin Core, DCAT, Schema.org and Ecological Trait Data Standard (Suppl. material 1). In case of missing properties in those standards, we created object and datatype properties using a dedicated nanopublication template. For the domain class, we created a nanopublication-based class "biodiversity monitoring schema". For the range class, we reused wherever possible existing OWL classes from OBO Foundry like OBI and ENVO, but in many cases, we had to create our own classes with nanopublications (Suppl. material 1). Nanopublications are small knowledge graphs with assertion, provenance and publication metadata (Kuhn et al. 2021).

Most metadata fields have a help text describing what type of metadata entry (response) is required. Response type can be number, text, URL, email, controlled lists or yes/no. All controlled lists are composed of terms and definitions from the thesauri used by the Integrated European Long-Term Ecosystem, critical zone and socio-ecological Research (eLTER) community, defined as skos:Concepts (Suppl. material 1). For the majority of the questions whose response types are controlled lists, users have the possibility to provide free text entries, in case the required value is not found in the drop-down. This allows new terms to be included in the controlled lists in the next versions of the template. Yet, some questions allow for multiple answers which can be added by using the plus icon. Moreover, it contains a field that allows us to link the metadata to the actual dataset and can be reused by other communities and adapted to particular project needs.

In the CEDAR environment, the FAIR-EuMon metadata template is located under the link "*Community Folders*", in the folder "*Shared*", sub-folder "*ADVANCE*". After completing all questions, the filled-out form turns into a metadata instance that can be saved and stored in the ADVANCE folder, as well as copied to clipboard as JSON-LD and RDF formats. If saved in the folder, it will be automatically named *FAIR-EuMon metadata template metadata*. We recommend rename it by providing a meaningful title, for example, the title of the dataset this metadata instance describes. To do so, click the three dots located on the right side of the title and choose *Rename*. An example of a filled-out form (metadata instance) is available under the folder ADVANCE, named as *Amphibian monitoring in German coal mines.*

We declared all technology choices to implement each of the FAIR Guiding Principles by means of a FAIR Implementation Profile (FIP; Schultes et al. (2020)). The FAIR-EuMon metadata schema and ADVANCE FIP (Suppl. material 2) are published as machine-readable formats via nanopublications and qualified by the GO FAIR Foundation, encouraging its reuse by other communities, and driving convergence on FAIR implementation choices.

**FAIR-EuMon metadata elements and fields**

The FAIR-EuMon metadata schema has been revised by marine, freshwater and terrestrial experts from UFZ, AWI, and by biodiversity monitoring experts from the German National Monitoring Centre for Biodiversity (NMZB) and National Research Data Infrastructure for Biodiversity (NFDI4Biodiversity). The entry form is composed of 43 questions, divided into 10 sections as described below.

1. SECTION: Read & Understood

This section provides general instructions on how to fill out the ADVANCE metadata templateV3 form.

2. SECTION: Monitoring Scheme Identification

In this section, users provide information that identifies their monitoring schemes.

2.1. Monitoring scheme title

The title by which the monitoring scheme is known should be provided.

2.2. Monitoring programme title

If the monitoring scheme is part of a larger programme (a set of monitoring schemes organised within the same institution), programme title should be provided.

2.3. Description

A brief summary with the most important details summarising the data (e.g. objectives, target group, key aspects, design, methods) should be provided.

2.4. Online locator

If data are published, the persistent identifier (e.g. DOI) of the data should be provided. Otherwise, a link to the data or to additional information about the data may be provided.

2.5. Keywords

At least three (3) keywords chosen from the drop-down list should be provided.

2.5.1. Please, provide suitable keywords if not found in the drop-down list.

If keywords are not found, users can type suitable keywords themselves.

3. SECTION: Contact Information

In this section, users provide contact details of organisations and people responsible for monitoring schemes

3.1. Responsible organisation

The ROR (Research Organization Registry) identifier of the responsible organisation should be provided. Otherwise, the link to the responsible organisation website can be given. ROR is a global, community-led registry of open persistent identifiers for research organisations.

3.2. Organisation type

The type of organisation (e.g. government, NGO, research centre) should be chosen from the drop-down list.

3.2.1. Please, provide organisation type if not found in the drop-down list.

Users can type their answer in case it is not found in the drop-down list.

3.3. Contact person full name

Users should provide the full name of the person responsible for the data.

3.4. Role

The role played by the responsible organisation (e.g. owner, contributor)

3.5. E-mail address

E-mail address of the contact person should be provided.

3.6. ORCID ID

The ORCID ID of the contact person should be provided. The ORCID (Open Researcher and Contributor) ID is a unique, persistent digital identifier for researchers.

4. SECTION: Funding

In this section, users declare funding sources of monitoring schemes.

4.1. Funding source

Users should provide the type of funding source (e.g. national, regional, private).

4.1.1. Please provide funding source if not found in the drop-down list.

Users can type source of funding if not found in the provided drop-down list.

4.2. Funding agency

The ROR ID of the funding agency should be provided.

5. SECTION: Intellectual Rights

In this section, users declare data accessibility.

5.1. Data availability

Users should choose from the drop-down list a statement about the data availability.

5.1.1 If data availability is restricted, please describe the conditions under which data might become available.

If “under certain conditions” is chosen, then a description on how to access the data should be provided.

5.2 Licence

Creative Commons (CC) licence types may be chosen from the drop-down list.

5.2.1. If your data are available under licences other than CC, please provide the applicable one.

This field allows users to type data access licences other than CC.

6. SECTION: Monitoring Scheme Information

In this section, users provide specific information related to the monitoring scheme goals and functioning.

6.1. Motivation to launch monitoring scheme

Users can choose their motivation to start the monitoring scheme in the drop-down list (e.g. national law, scientific interest, impact assessment).

Please provide your motivation if not found in the drop-down list.

Users have the possibility to type their motivation other than those provided in the drop-down list.

6.2. Scope of monitoring

Users can choose the context of the monitoring scheme (e.g. distribution trend, community structure, physical-chemical environment).

6.2.1. Please provide the scope of monitoring if not found in the drop-down list.

Users can provide scope of monitoring other than those available in the drop-down list.

6.3. Biodiversity threats and pressures addressed

A list of biodiversity threats and pressures (e.g. climate change, invasive species, habitat loss and fragmentation) is provided from which users can choose. Definitions can be found at EnvThes.

6.3.1. Please provide threat or pressure addressed if not found in the drop-down list.

Users can type other threats or pressures addressed not provided in the list.

6.4. Type of data collected

A list of data types (e.g. presence-absence, count) is provided for users to choose from. Definitions are provided at EnvThes.

6.4.1. Please provide type of data collected if not found in the drop-down list.

Users can provide other types of data collected other than those found in the drop-down list.

6.5.Training / expert knowledge required to take part in data collection

This is a yes / no question whether training or expert knowledge is required to participate in data collection.

6.6.Number of professionals involved

Number of people who have a professional, job-related interest in the monitoring and who receive their main salary from monitoring and related activities; professionals will generally have special training or monitoring expertise in their professional capacity.

6.7. Number of volunteers involved

Number of people who participate in monitoring in their spare time, not having monitoring as their main income, but who may receive some economic compensation for participating; volunteers may have considerable expertise in monitoring-related fields but generally not related to their profession. In case of uncertainty, please provide an estimated mean number of volunteers involved.

6.8. Staff costs

Users provide estimated staff costs (salaries) per year to run monitoring activities. Euro should be used as currency.

6.9.Monitoring activities costs

Users provide estimated costs per year to run monitoring activities, except of salaries (e.g. travel, fieldwork, lab work, office work etc.). Euro should be used as currency.

7. SECTION: Temporal Coverage

In this section, users provide starting and ending year of data collection.

7.1. Start year

The year in which monitoring activities started should be provided.

7.2. End year

The year in which monitoring activities finished should be provided. Should be filled only if monitoring activities have already finished or if ending year is planned.

8. SECTION: Spatial Coverage

In this section, users provide information about the spatial coverage of monitoring schemes.

8.1. Geographical level

The geographical level to which the monitoring results or conclusions can be applied (e.g. local, national).

8.2. Country

Countries where monitoring activities take place should be provided.

8.3. Total area

Area (in km^2^) to which results can be extrapolated (e.g. inhabited area of a population, park area for monitoring restricted to a park, country area for national monitoring).

8.4. Altitudinal range

Range of elevation (minimum and maximum values) above sea level of the study site should be provided in metres.

8.4.1. Altitude (Min. value)

Minimum altitude in metres.

8.4.2. Altitude (Max. value)

Maximum altitude in metres.

8.5. Sampling sites located in protected areas

Users can state whether sampling sites are totally, partially or not located in protected areas.

8.6. Biogeographical region

Biogeographical and marine regions of Europe can be chosen in the drop-down list. Users can use the Natura 2000 Network Viewer (under “Layers”, select “Biogeographical regions”) to view the European terrestrial biogeographic regions and assign the corresponding region to their study areas.

8.7. Habitat type

Habitat classifications from different sources are provided in three drop-down lists: the CORINE biotopes, the EUNIS habitat classification and the Habitats Directive Annex I. Users can choose their preferred classification schema.

8.7.1. CORINE habitat classification

Users select from this list if they prefer CORINE biotopes for habitat classification.

8.7.2. EUNIS habitat classification

Users select from this list if they prefer EUNIS biotopes for habitat classification.

8.7.3. Habitats Directive classification

Users select from this list if they prefer Habitats Directive Annex I for habitat classification.

8.7.4. Please provide habitat type and classification scheme if not found in the drop-down lists.

Users can use this field to type other preferred habitat classifications (e.g. national classifications).

9. SECTION: Taxonomic Coverage

In this section, users provide information about taxonomic coverage of monitoring schemes.

9.1. Organism group

Drop-down lists of the most common monitored organisms in Europe are provided below.

9.1.1. Birds

9.1.1.1. Please provide group of birds if not found in the drop-down list.

9.1.2. Mammals

9.1.2.1. Please provide group of mammals if not found in the drop-down list.

9.1.3. Other vertebrates

9.1.4. Invertebrates

9.1.4.1. Please provide group of invertebrates if not found in the drop-down list.

9.1.5. Plants

9.1.5.1. Please provide group of plants if not found in the drop-down list.

9.1.6. Other organisms

9.1.7. Please, provide group of organisms if not found in the drop-down list.

Users can still type other monitored organisms not available in the provided drop-down lists.

9.2. Species of community interest

Species of community interest are listed in appendices of European Directives or under any legal text in Europe (e.g. CITES, CMS). If species of community interest are monitored, users should provide species’ scientific names and respective URLs.

9.2.1. Species’ scientific name

If species of community interest are monitored, users should copy and paste species’ scientific names from the GBIF database (e.g. *Lacertaviridis*).

9.2.2. Species URL

If species of community interest are monitored, users should copy and paste species URLs from the GBIF database (e.g. https://www.gbif.org/species/6159273).

10. SECTION: Methods and Sampling Information

In this last section, users provide relevant information about the methods used for collecting data.

10.1. Experimental design

A list of possible experimental designs (e.g. control treatments, before-after comparison) is provided from which users can choose.

10.2. Site selection

Users state how sampling sites have been selected. A drop-down list is provided (e.g. random sampling, systematic sampling).

10.2.1. Please, describe how sites are selected if not found in the drop-down list.

Users can still type how sampling sites have been selected.

10.3. Number of sampling sites

Users provide information about the number of sampling sites.

10.4. Sampling duration

Users provide information about time spent per sampling site during a single visit. Time unit should be added by users (e.g. hours, days).

10.5. Frequency of monitoring

Users provide information about the number of visits to a sampling site within a particular period (e.g. once a month, twice a year).

10.6. Changes in monitoring procedure during monitoring scheme lifetime

This is a yes / no question whether changes in monitoring procedures have taken place during the monitoring scheme’s lifetime.

10.6.1. If yes, please describe changes.

Users describe changes in monitoring procedure.

10.7. Field methods

In this last field, users describe methods used to collect data.

**Conclusion and Outlook**

The ADVANCE project provided a FAIR metadata schema for the biodiversity monitoring community to allow comprehensive descriptions of biodiversity monitoring schemes across marine, terrestrial and freshwater realms. The schema is implemented as a CEDAR template to provide a human-readable form that can be filled out as is and also re-used to comply with other projects' needs. Moreover, this metadata schema is machine-interoperable, making use of persistent identifiers, structured vocabularies and ontologies to FAIRify metadata about biodiversity monitoring schemes. Being represented in JSON-LD, the generated metadata instances can be exposed as FAIR data on a Web portal to finally allow semantic faceted search across all monitoring schemes, based on their described attributes.

The FAIR-EuMon metadata schema will replace the former EuMon questionnaire and provide the framework for making the EuMon database publicly available again. Currently available on CEDAR, the metadata form can already be filled out to add new biodiversity monitoring schemes. In the meantime, we are working on providing public access to the EuMon database, using the FAIR-EuMon metadata schema. This will foster biodiversity trend analyses by providing FAIR data across marine, freshwater and terrestrial realms.

The EUMon database has been included in various discussions for promoting the establishment of a National Monitoring Centre for Biodiversity in Germany (NMZB; Henle (2012), K?hl et al. (2020)). We plan to jointly use and expand the database for the German biodiversity monitoring landscape. If current recommendations for the establishment of a European biodiversity monitoring coordination institution (Liquete et al. 2024) turns into reality, the FAIR-EuMon database can provide valuable assets to overcome the highly fragmented European biodiversity monitoring landscape. Our vision is that the European biodiversity community maintains and regularly updates these assets as a common heritage for the benefit of biodiversity conservation from the local to the European scale.

### Audience

Biodiversity monitoring community

## Supplementary Material

D6A991B3-6AA6-5144-AF53-C6CAB9B63467 10.3897/BDJ.12.e125132.suppl1 Supplementary material 1 The FAIR-EuMon metadata template characteristics Data type excel file Brief description A description of the FAIR-EuMon metadata template is given. All fields refer to properties from ontologies having a Globally Unique, Persistent, Resolvable Identifier (GUPRI). All controlled lists are composed by terms of the Thesauri used by the Integrated European Long-Term Ecosystem, critical zone and socio-ecological Research (eLTER) community. File: oo_1084630.xlsx https://binary.pensoft.net/file/1084630 Menger et al

CD1B1B10-A713-5D51-A814-F50D081A724D 10.3897/BDJ.12.e125132.suppl2 Supplementary material 2 ADVANCE FIP Data type excel file Brief description All technology choices to implement each of the FAIR Guiding Principles are declared by means of a FAIR Implementation Profile (FIP). File: oo_1084916.xlsx https://binary.pensoft.net/file/1084916 Menger et al

## Figures and Tables

**Table:** 

Nanopublication	Creator	Date
https://w3id.org/kpxl/pensoft/bdj/np/RAtuzc76l2n2_q4j6ORAT9WQRwMRoqYi1LFpATyRdZaTo	0000-0002-6968-8275	01-08-2024 06:52:27
https://w3id.org/kpxl/pensoft/bdj/np/RA0RTUCJb2Ggf4_Jx7YGgwMUMSDJFFFYKjv94jxDsLizA	0000-0002-6968-8275	01-08-2024 06:53:33
